# Applicability of pulse pressure variation: how many shades of grey?

**DOI:** 10.1186/s13054-015-0869-x

**Published:** 2015-03-25

**Authors:** Frederic Michard, Denis Chemla, Jean-Louis Teboul

**Affiliations:** Edwards Lifesciences, 1 Edwards Way, Irvine, CA USA; Physiology department-INSERM U999, CHU de Bicêtre, Université Paris Sud, 78 rue du Général Leclerc, 94270 le Kremlin Bicêtre, France; Medical ICU, CHU de Bicêtre, Université Paris Sud, 78 rue du Général Leclerc, 94270 le Kremlin Bicêtre, France

Since its first description in 1999 [1], many studies have demonstrated the value of pulse pressure variation (PPV) as a predictor of fluid responsiveness. These studies were pooled together in a recent meta-analysis [2] concluding that PPV predicts fluid responsiveness accurately (sensitivity 88%, specificity 89%), so long as limitations to its use [3,4] are understood and respected (Figure 1).

**Figure 1:**
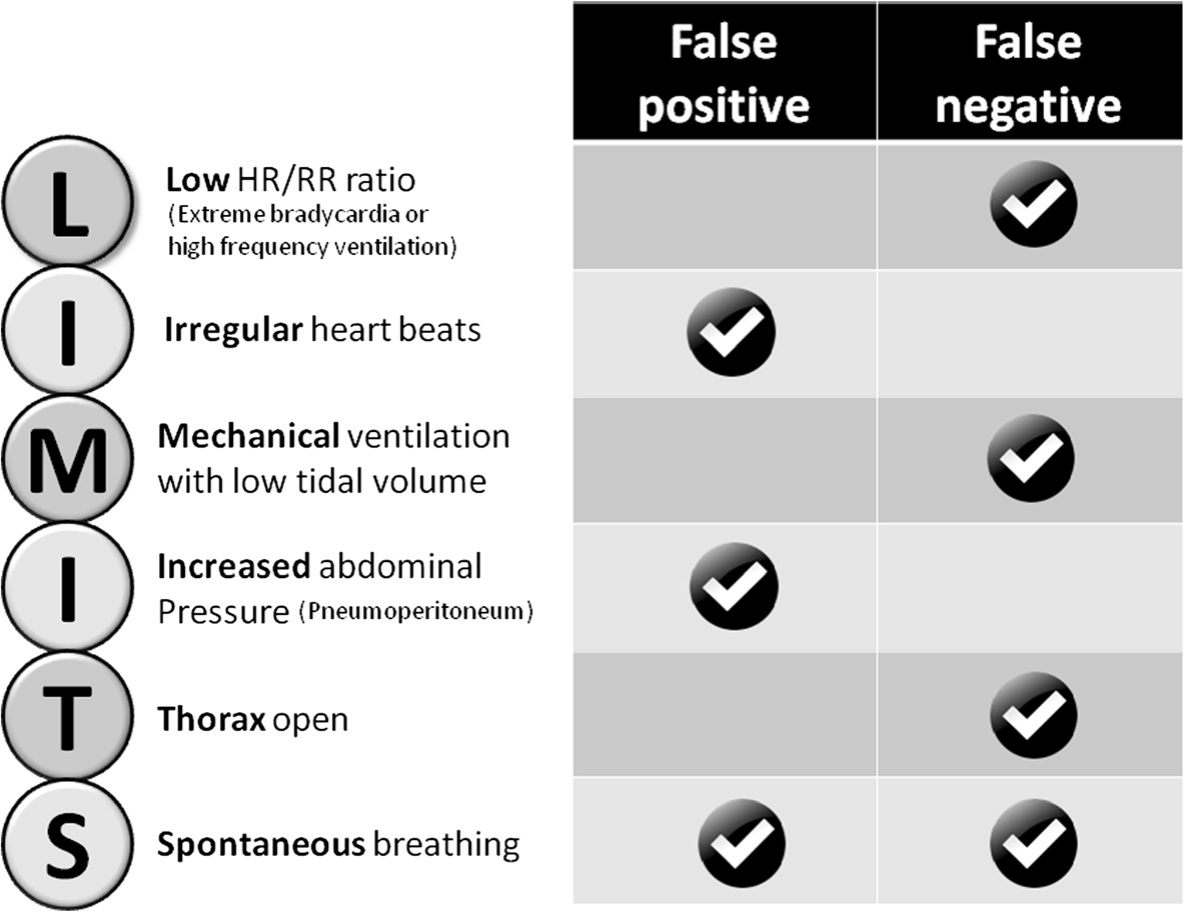
**Most common physiological limitations to the use of pulse pressure variation can be summarized as ‘LIMITS’.** HR/RR, heart rate/respiratory rate.

## The applicability of pulse pressure variation

Several studies have quantified the proportion of patients in whom PPV can be used as a predictor of fluid responsiveness [5-7]. Logically, the applicability is higher in the operating theatre than in the ICU, because limitations are less often encountered [8,9]. There is currently a trend towards a reduction in tidal volume, not only in ICU patients with acute lung injury, but also in patients with healthy lungs undergoing surgery. Futier and colleagues [10] showed that a tidal volume of 6 ml/kg during surgery is associated with a better post-surgical outcome than a tidal volume of 11 ml/kg. However, nothing indicates that 6 ml/kg is better than 8 ml/kg. Actually, a recent comparison between tidal volume and outcome done on 29,343 patients who underwent general anesthesia with mechanical ventilation suggests that the ideal tidal volume is somewhere between 8 and 10 ml/kg [11]. Ultimately, the applicability of PPV depends on case mix (whether patients are mechanically ventilated, and whether they have arrhythmia), and on clinicians beliefs and practice (do they prefer ventilating their patients with 6 or 8 ml/kg?). It may easily vary from 0% (extubated patients) to 99% (typical open colorectal or hip fracture patient ventilated with 8 ml/kg) [8].

## The zone of uncertainty, also called the grey zone

**Figure 2:**
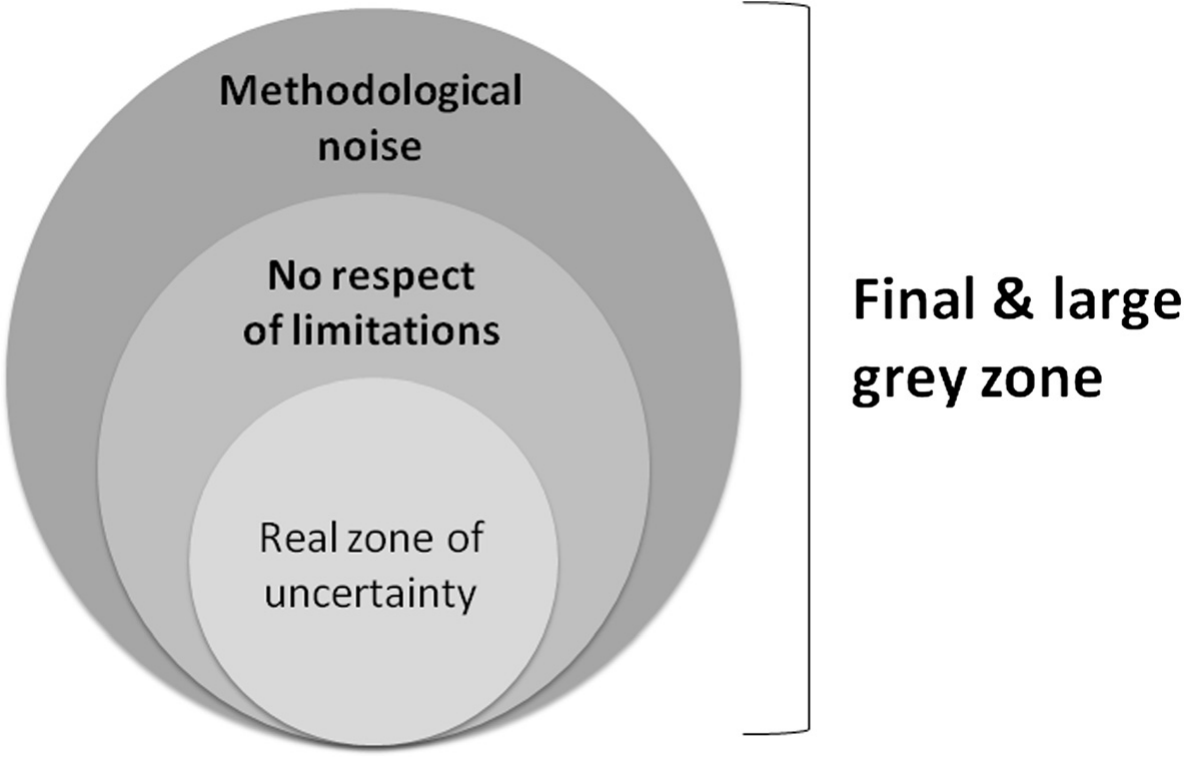
**Not respecting pulse pressure variation limitations and methodological noise artificially increase the zone of uncertainty, also called the grey zone.**

Cannesson and colleagues [12], and more recently Biais and colleagues [13], have used the ‘grey zone’ approach to investigate the clinical value of PPV. The concept has practical value because it allows the determination of three zones: a zone where PPV predicts a positive response to fluid loading, a zone where PPV predicts a negative response, and a third zone of uncertainty or ‘grey zone’. This approach should be used exclusively to assess the intrinsic predictive value of PPV, once limitations to its use have been discarded. Unfortunately, when assessing their grey zone, both Cannesson and colleagues [12] and Biais and colleagues [13] have analyzed many measurements coming from patients ventilated with a small tidal volume, or with a low heart rate/respiratory rate ratio. Because PPV does not work well in this context, their grey zones were artificially extended. In this respect, Biais and colleagues [13] showed in a subgroup analysis that the grey zone was larger in patients with a low tidal volume than in patients with a tidal volume of at least 8 ml/kg, and clearly acknowledged that ‘the wide range of tidal volume can explain the importance of the grey zone and the variation of grey zone values among centers’. Both Cannesson and colleagues [12] and Biais and colleagues [13] also pooled data from studies where different techniques were used to measure cardiac output (CO). Cannesson and colleagues [12] mentioned that they ‘classified responder and non-responder patients using various methods of CO measurements, all of which have unique errors of measurements and limited clinical agreement between them’, suggesting that a responder with one method could have been classified as a non-responder by another method [14]. Biais and colleagues [13] acknowledged that ‘the methods of CO measurements were not uniform and this may have extended the grey zone’. Therefore, from a methodological standpoint, the grey zones in both studies [12,13] were undoubtedly enlarged by these confounding factors, or shades of grey… and readers were left in the dark with regard to the real zone of uncertainty for PPV (Figure 2).

## The limits of the ‘responders versus non-responders’ binary approach

In daily practice, it is at least as important to have a predictor of the amount of the increase in CO induced by fluid loading as knowing if CO will increase by more or less than 15%. What is the clinically relevant difference between two patients increasing their CO by 14 and 16%, respectively? Studies have repeatedly documented a linear and positive relationship between PPV before fluid administration and the percentage increase in CO in response to fluid loading [1-3,15]. This means that, in the presence of an intermediate PPV value - that is, within the grey zone - one may expect a mild increase in CO. This is not minor information when assessing the benefit/risk ratio of fluid therapy.

## Conclusion

Recent studies about the applicability of PPV [5-7], or the study from Biais and colleagues [13] reporting a large grey zone, may lead to the wrong conclusion that PPV has limited clinical value. Several randomized controlled trials have investigated whether fluid management based on PPV (or on surrogate parameters) may improve patients’ outcomes. A recent meta-analysis [16] of these trials showed that PPV-based fluid management is associated with a significant decrease in post-surgical morbidity and length of stay. In other words, PPV-based strategies have the potential to improve quality of care and decrease health care costs at the same time [17]. For these reasons, clinicians, who have already embraced the concept widely [18,19], are now encouraged to use PPV (and surrogate parameters) in an attempt to make more rational and informed decisions regarding fluid management [20,21].

## Abbreviations

CO: Cardiac output
PPV: Pulse pressure variation
